# Supplementary material for: Limited dispersal and an unexpected aggression pattern in a native supercolonial ant
Source: Ecol Evol. 2020 Mar 13;10(8):3671–85. doi: 10.1002/ece3.6154 (PMC7160175; doi:10.1002/ece3.6154)
Supplement: Supplementary file 1 — Tables S1‐S6 [file ECE3-10-3671-s001.docx]

**“Limited dispersal and an unexpected aggression pattern in a native supercolonial ant”**

S. M. Hakala, M. Ittonen, P. Seppä, H. Helanterä

Supplementary material: Details on microsatellite data

**Supplementary table 1**

Properties of the microsatellite data when the supercolony field Särkkilen is treated as one population (A), and when it is treated as two populations divided by a nestless gap in the middle (B). N = the number of samples from a site, N_A_ = the mean of the number of alleles per microsatellite marker for each site, AR = mean allelic richness, H_o_ = observed heterozygosity, H_e_ = expected heterozygocity, F_IS_ = inbreeding coefficient, F_ST_ = the mean of the pairwise F_ST_ comparisons with other sites.

| A |  |  |  |  |  |  |  |
| --- | --- | --- | --- | --- | --- | --- | --- |
| Site | *N* | N_A_ | AR | H_h_ | H_e_ | F_IS_ | F_ST_ |
| Särkkilen | 233 | 8.2 | 4.3 | 0.72 | 0.74 | 0.031 | 0.038 |
| Storsand | 16 | 4.6 | 3.6 | 0.67 | 0.66 | –0.005 | 0.058 |
| Lillgård | 7 | 4.1 | 3.5 | 0.60 | 0.59 | 0.002 | 0.066 |
| Storgård | 29 | 6.1 | 4.0 | 0.70 | 0.71 | 0.021 | 0.042 |
|  |  |  |  |  |  |  |  |
| Site | *N* | N_A_ | AR | H_h_ | H_e_ | F_IS_ | F_ST_ |
| Särkkilen I & II | 122 | 7.9 | 4.2 | 0.71 | 0.73 | 0.028 | 0.044 |
| Särkkilen III & IV | 111 | 7.6 | 4.3 | 0.72 | 0.74 | 0.016 | 0.060 |
| Storsand | 16 | 4.6 | 3.6 | 0.67 | 0.66 | –0.005 | 0.061 |
| Lillgård | 7 | 4.1 | 3.5 | 0.60 | 0.59 | 0.002 | 0.068 |
| Storgård | 29 | 6.1 | 4.0 | 0.70 | 0.71 | 0.021 | 0.046 |

**Supplementary table 2**

Allele frequences for each marker in each subpopulation. The number of succesfully genotyped samples for each locus in each popualtion are in parentheses. We did not get any data for the marker Fe17, and did not use the markers Fe11, Fe21 and P22 in our analyses due to heterozygote deficiency or missing data.

| Marker |  | Field (the number of succesfully genotyped samples in parentheses) | | | |
| --- | --- | --- | --- | --- | --- |
| Fe13 | Allele | Särkkilen ( 224) | Storsand (16) | Lillgård (7) | Storgård (29) |
|  | 149 | 0.161 | 0.469 | 0.000 | 0.310 |
|  | 164 | 0.192 | 0.438 | 0.357 | 0.207 |
|  | 211 | 0.076 | 0.063 | 0.143 | 0.034 |
|  | 226 | 0.065 | 0.000 | 0.000 | 0.000 |
|  | 229 | 0.004 | 0.000 | 0.000 | 0.017 |
|  | 232 | 0.022 | 0.031 | 0.214 | 0.155 |
|  | 235 | 0.022 | 0.000 | 0.000 | 0.000 |
|  | 240 | 0.009 | 0.000 | 0.000 | 0.052 |
|  | 243 | 0.239 | 0.000 | 0.000 | 0.121 |
|  | 246 | 0.203 | 0.000 | 0.214 | 0.086 |
|  | 249 | 0.000 | 0.000 | 0.071 | 0.000 |
|  | 252 | 0.007 | 0.000 | 0.000 | 0.017 |
| Fe19 | Allele | Särkkilen (221) | Storsand (15) | Lillgård (7) | Storgård (29) |
|  | 186 | 0.036 | 0.000 | 0.000 | 0.000 |
|  | 190 | 0.276 | 0.200 | 0.429 | 0.483 |
|  | 192 | 0.346 | 0.400 | 0.286 | 0.345 |
|  | 194 | 0.179 | 0.067 | 0.071 | 0.086 |
|  | 198 | 0.158 | 0.333 | 0.000 | 0.086 |
|  | 200 | 0.005 | 0.000 | 0.214 | 0.000 |
| Fe42 | Allele | Särkkilen (220) | Storsand (15) | Lillgård (5) | Storgård (28) |
|  | 272 | 0.589 | 0.800 | 0.800 | 0.500 |
|  | 274 | 0.327 | 0.200 | 0.200 | 0.446 |
|  | 276 | 0.025 | 0.000 | 0.000 | 0.000 |
|  | 278 | 0.059 | 0.000 | 0.000 | 0.054 |
| Fe51 | Allele | Särkkilen (220) | Storsand (14) | Lillgård (7) | Storgård (29) |
|  | 85 | 0.002 | 0.000 | 0.000 | 0.000 |
|  | 89 | 0.050 | 0.107 | 0.000 | 0.103 |
|  | 92 | 0.159 | 0.179 | 0.143 | 0.155 |
|  | 94 | 0.332 | 0.107 | 0.571 | 0.517 |
|  | 96 | 0.361 | 0.321 | 0.071 | 0.121 |
|  | 98 | 0.095 | 0.286 | 0.214 | 0.103 |
|  |  |  |  |  |  |
| Fy4 | Allele | Särkkilen (189) | Storsand (12) | Lillgård (6) | Storgård (22) |
|  | 277 | 0.344 | 0.583 | 0.083 | 0.500 |
|  | 279 | 0.286 | 0.333 | 0.083 | 0.295 |
|  | 281 | 0.370 | 0.083 | 0.833 | 0.205 |
| Fe16 | Allele | Särkkilen (173) | Storsand (16) | Lillgård (6) | Storgård (28) |
|  | 166 | 0.055 | 0.000 | 0.083 | 0.125 |
|  | 172 | 0.009 | 0.000 | 0.000 | 0.000 |
|  | 177 | 0.003 | 0.031 | 0.000 | 0.000 |
|  | 178 | 0.049 | 0.000 | 0.000 | 0.036 |
|  | 179 | 0.035 | 0.000 | 0.000 | 0.143 |
|  | 180 | 0.026 | 0.000 | 0.000 | 0.018 |
|  | 181 | 0.006 | 0.000 | 0.000 | 0.000 |
|  | 183 | 0.061 | 0.000 | 0.000 | 0.036 |
|  | 184 | 0.052 | 0.125 | 0.000 | 0.000 |
|  | 185 | 0.124 | 0.156 | 0.000 | 0.125 |
|  | 186 | 0.046 | 0.188 | 0.000 | 0.161 |
|  | 187 | 0.231 | 0.219 | 0.250 | 0.179 |
|  | 188 | 0.038 | 0.000 | 0.083 | 0.000 |
|  | 189 | 0.049 | 0.094 | 0.583 | 0.054 |
|  | 190 | 0.049 | 0.063 | 0.000 | 0.018 |
|  | 191 | 0.147 | 0.125 | 0.000 | 0.089 |
|  | 192 | 0.017 | 0.000 | 0.000 | 0.000 |
|  | 193 | 0.003 | 0.000 | 0.000 | 0.018 |
| Fy7 | Allele | Särkkilen (210) | Storsand (16) | Lillgård (6) | Storgård (29) |
|  | 239 | 0.076 | 0.000 | 0.000 | 0.034 |
|  | 241 | 0.221 | 0.344 | 0.500 | 0.345 |
|  | 243 | 0.052 | 0.000 | 0.000 | 0.000 |
|  | 245 | 0.043 | 0.156 | 0.167 | 0.000 |
|  | 247 | 0.126 | 0.125 | 0.167 | 0.034 |
|  | 249 | 0.021 | 0.000 | 0.000 | 0.017 |
|  | 251 | 0.017 | 0.000 | 0.000 | 0.000 |
|  | 252 | 0.005 | 0.000 | 0.000 | 0.000 |
|  | 254 | 0.326 | 0.344 | 0.167 | 0.431 |
|  | 258 | 0.043 | 0.031 | 0.000 | 0.034 |
|  | 260 | 0.036 | 0.000 | 0.000 | 0.000 |
|  | 310 | 0.002 | 0.000 | 0.000 | 0.086 |
|  | 312 | 0.017 | 0.000 | 0.000 | 0.000 |
|  | 314 | 0.014 | 0.000 | 0.000 | 0.017 |
| Fe37 | Allele | Särkkilen (144) | Storsand (16) | Lillgård (5) | Storgård (23) |
|  | 116 | 0.021 | 0.000 | 0.000 | 0.000 |
|  | 118 | 0.139 | 0.156 | 0.100 | 0.130 |
|  | 127 | 0.010 | 0.000 | 0.000 | 0.217 |
|  | 129 | 0.493 | 0.375 | 0.700 | 0.174 |
|  | 131 | 0.125 | 0.125 | 0.100 | 0.239 |
|  | 133 | 0.135 | 0.281 | 0.100 | 0.239 |
|  | 135 | 0.076 | 0.063 | 0.000 | 0.000 |
| P22 | Allele | Särkkilen (222) | Storsand (16) | Lillgård (6) | Storgård (28) |
|  | 131 | 0.009 | 0.000 | 0.000 | 0.000 |
|  | 141 | 0.005 | 0.000 | 0.000 | 0.000 |
|  | 146 | 0.034 | 0.000 | 0.000 | 0.000 |
|  | 148 | 0.489 | 0.438 | 0.667 | 0.536 |
|  | 150 | 0.412 | 0.281 | 0.250 | 0.464 |
|  | 152 | 0.052 | 0.281 | 0.083 | 0.000 |
| Fe38 | Allele | Särkkilen (218) | Storsand (16) | Lillgård (7) | Storgård (27) |
|  | 66 | 0.041 | 0.000 | 0.143 | 0.019 |
|  | 70 | 0.064 | 0.000 | 0.000 | 0.000 |
|  | 72 | 0.209 | 0.281 | 0.214 | 0.259 |
|  | 74 | 0.005 | 0.031 | 0.000 | 0.037 |
|  | 78 | 0.218 | 0.281 | 0.286 | 0.259 |
|  | 80 | 0.069 | 0.063 | 0.071 | 0.111 |
|  | 82 | 0.124 | 0.156 | 0.071 | 0.167 |
|  | 84 | 0.241 | 0.156 | 0.071 | 0.019 |
|  | 87 | 0.030 | 0.031 | 0.143 | 0.130 |
| Fl21 | Allele | Särkkilen (220) | Storsand (14) | Lillgård (7) | Storgård (28) |
|  | 211 | 0.384 | 0.536 | 0.143 | 0.339 |
|  | 213 | 0.086 | 0.000 | 0.357 | 0.054 |
|  | 221 | 0.177 | 0.143 | 0.286 | 0.232 |
|  | 223 | 0.352 | 0.321 | 0.214 | 0.375 |
| Fe11 | Allele | Särkkilen (95) | Storsand (1) | Lillgård (0) | Storgård (9) |
|  | 135 | 0.221 | 0.000 | 0.000 | 0.111 |
|  | 136 | 0.095 | 0.000 | 0.000 | 0.056 |
|  | 139 | 0.079 | 1.000 | 0.000 | 0.444 |
|  | 140 | 0.458 | 0.000 | 0.000 | 0.333 |
|  | 141 | 0.142 | 0.000 | 0.000 | 0.056 |
|  | 142 | 0.005 | 0.000 | 0.000 | 0.000 |
|  |  |  |  |  |  |
| Fe21 | Allele | Särkkilen (92) | Storsand (0) | Lillgård (0) | Storgård (20) |
|  | 82 | 0.011 | 0.000 | 0.000 | 0.000 |
|  | 98 | 0.293 | 0.000 | 0.000 | 0.000 |
|  | 100 | 0.185 | 0.000 | 0.000 | 0.000 |
|  | 102 | 0.429 | 0.000 | 0.000 | 0.000 |
|  | 104 | 0.065 | 0.000 | 0.000 | 0.000 |
|  | 106 | 0.005 | 0.000 | 0.000 | 0.000 |
|  | 108 | 0.011 | 0.000 | 0.000 | 0.000 |

**Supplementary table 3**

The number of alleles in each subpopulation (A), observed heterozygosity (H_o_) and expected heterozygosity (H_e_) under Hardy-Weinberg equilibrium, and a P value for Fisher’s exact test for deviation from Hardy-Weinberg equilibrium (p). P values lower than 0.05 are in bold. We did not get any data for the marker Fe17, and did not use the markers Fe11, Fe21 and P22 in our analyses due to heterozygote deficiency or missing data.

|  | Särkkilen | | | | Storsand | | | | Lillgård | | | | Storgård | | | |
| --- | --- | --- | --- | --- | --- | --- | --- | --- | --- | --- | --- | --- | --- | --- | --- | --- |
| Marker | A | H_o_ | H_e_ | p | A | H_o_ | H_e_ | p | A | H_o_ | H_e_ | p | A | H_o_ | H_e_ | p |
| Fe13 | 11 | 0.804 | 0.828 | 0.714 | 4 | 0.500 | 0.584 | 0.2968 | 5 | 0.714 | 0.755 | 0.466 | 9 | 0.828 | 0.810 | 0.598 |
| Fe19 | 6 | 0.579 | 0.746 | **0.000** | 4 | 0.800 | 0.684 | 0.6362 | 4 | 0.571 | 0.684 | 0.273 | 4 | 0.655 | 0.633 | 0.693 |
| Fe42 | 4 | 0.559 | 0.542 | 0.289 | 2 | 0.267 | 0.320 | 0.4604 | 2 | 0.400 | 0.320 | 1.000 | 3 | 0.429 | 0.548 | 0.349 |
| Fe51 | 6 | 0.736 | 0.722 | 0.814 | 5 | 0.786 | 0.760 | 0.663 | 4 | 0.714 | 0.602 | 0.627 | 5 | 0.552 | 0.672 | **0.015** |
| Fy4 | 3 | 0.603 | 0.663 | 0.217 | 3 | 0.500 | 0.542 | 0.6023 | 3 | 0.167 | 0.292 | 0.084 | 3 | 0.591 | 0.621 | 0.297 |
| Fe16 | 18 | 0.780 | 0.887 | **< 0.0001** | 8 | 0.813 | 0.848 | 0.6628 | 4 | 0.667 | 0.583 | 0.762 | 12 | 0.857 | 0.876 | 0.378 |
| Fy7 | 14 | 0.962 | 0.814 | **< 0.0001** | 5 | 1.000 | 0.723 | 0.0576 | 4 | 0.667 | 0.667 | 0.312 | 8 | 0.897 | 0.684 | **0.028** |
| Fe37 | 7 | 0.708 | 0.697 | 0.804 | 5 | 0.688 | 0.736 | 0.4845 | 4 | 0.400 | 0.480 | 0.321 | 5 | 0.739 | 0.791 | 0.910 |
| P22 | 6 | 0.167 | 0.587 | **< 0.0001** | 3 | 0.125 | 0.650 | **< 0.0001** | 3 | 0.333 | 0.486 | 0.506 | 2 | 0.071 | 0.497 | **< 0.0001** |
| Fe38 | 9 | 0.807 | 0.824 | 0.488 | 7 | 0.875 | 0.787 | 0.3146 | 7 | 1.000 | 0.816 | 0.859 | 8 | 0.741 | 0.807 | 0.054 |
| Fl21 | 4 | 0.641 | 0.689 | 0.253 | 3 | 0.500 | 0.589 | 0.1217 | 4 | 0.714 | 0.724 | 0.340 | 4 | 0.714 | 0.688 | 0.116 |
| Fe11 | 6 | 0.526 | 0.706 | **< 0.0001** | 1 | 0 | 0 |  | 0 | 0 | 0 |  | 5 | 0.556 | 0.673 | 0.091 |
| Fe21 | 7 | 0.630 | 0.691 | 0.094 | 0 | 0 | 0 |  | 0 | 0 | 0 |  | 0 | 0 | 0.000 |  |

**Supplementary table 4**

Tests of heterozygote deficiency in each subpopulation. P values lower than 0.05 are in bold. We did not get any data for the marker Fe17, and did not use the markers Fe11, Fe21 and P22 in our analyses due to heterozygote deficiency or missing data.

|  | Särkkilen |  |  | Storsand | |  | Lillgård |  |  | Storgård |  |  |
| --- | --- | --- | --- | --- | --- | --- | --- | --- | --- | --- | --- | --- |
| Marker | p | SE | FIS | p | SE | FIS | p | SE | FIS | p | SE | FIS |
| Fe13 | 0.1266 | 0.02 | 0.0317 | 0.2782 | 0.0093 | 0.1753 | 0.342 | 0.0092 | 0.1304 | 0.6236 | 0.0211 | -0.0037 |
| Fe19 | **0.0005** | 0.00 | 0.2254 | 0.8607 | 0.0051 | -0.1351 | 0.27 | 0.0073 | 0.2381 | 0.6861 | 0.0089 | -0.0172 |
| Fe42 | 0.8518 | 0.01 | -0.0287 | 0.4604 | 0.0021 | 0.2 | 1 | 0 | -0.1429 | 0.1484 | 0.0047 | 0.2349 |
| Fe51 | 0.7915 | 0.02 | -0.0171 | 0.2685 | 0.0066 | 0.0035 | 0.8541 | 0.0054 | -0.1111 | 0.1354 | 0.006 | 0.1964 |
| Fy4 | **0.0366** | 0.00 | 0.0927 | 0.381 | 0.0059 | 0.12 | 0.0837 | 0.0035 | 0.5 | 0.2564 | 0.0046 | 0.0714 |
| Fe16 | **0** | 0.00 | 0.123 | 0.2657 | 0.0145 | 0.0736 | 0.7618 | 0.0069 | -0.0526 | 0.4076 | 0.0316 | 0.04 |
| Fy7 | 0.9907 | 0.01 | -0.1795 | 1 | 0 | -0.3559 | 0.2477 | 0.0059 | 0.0909 | 0.9977 | 0.0015 | -0.2954 |
| Fe37 | 0.1076 | 0.01 | -0.0124 | **0.0414** | 0.0032 | 0.0984 | 0.3211 | 0.0089 | 0.2727 | 0.1918 | 0.0067 | 0.0878 |
| P22 | **< 0.0001** | 0.00 | 0.7173 | **< 0.0001** | 0.0000 | 0.8187 | 0.2677 | 0.0047 | 0.3939 | **< 0.0001** | 0.0000 | 0.8612 |
| Fe38 | 0.2136 | 0.02 | 0.0227 | 0.6993 | 0.0137 | -0.0797 | 1 | 0.0000 | -0.1507 | 0.1568 | 0.0126 | 0.1003 |
| Fl21 | 0.0538 | 0.01 | 0.0727 | 0.2276 | 0.0038 | 0.1875 | 0.2729 | 0.0056 | 0.0909 | 0.0819 | 0.0037 | -0.0208 |
| Fe11 | **0** | 0.00 | 0.2595 |  |  |  |  |  |  | 0.2467 | 0.0101 | 0.2308 |
| Fe21 | 0.1511 | 0.02 | 0.0929 |  |  |  |  |  |  |  |  |  |

**Supplementary table 5**

Linkage disequilibrium for each pair of microsatellite markers in each subpopulation, estimated with Fisher’s method. Missing data are due to genotyping failures. P values lover than 0.05 are in bold. We did not get any data for the marker Fe17, and did not use the markers Fe11, Fe21 and P22 in our analyses due to heterozygote deficiency or missing data.

| All subpopulations | | | | Särkkilen | | | | Storsand | | | | | Lillgård | | | | | | Storgård | | | | | | |
| --- | --- | --- | --- | --- | --- | --- | --- | --- | --- | --- | --- | --- | --- | --- | --- | --- | --- | --- | --- | --- | --- | --- | --- | --- | --- |
| Markers | χ^2^ | df | p | Markers | χ^2^ | df | p | Markers | χ^2^ | df | | p | Markers | χ^2^ | df | | p | | Markers | | χ^2^ | df | | p | |
| Fe13 & Fe19 | 4.925 | 8 | 0.766 | Fe13 & Fe19 | 2.481 | 2 | 0.289 | Fe13 & Fe19 | 0.066 | 2 | 0.968 | | Fe13 & Fe19 | 0.000 | 2 | | 1.000 | | Fe13 & Fe19 | 2.646 | | 2 | | 0.266 | |
| Fe13 & Fe42 | 12.874 | 8 | 0.116 | Fe13 & Fe42 | 1.053 | 2 | 0.591 | Fe13 & Fe42 | 3.714 | 2 | 0.156 | | Fe13 & Fe42 | 0.000 | 2 | | 1.000 | | Fe13 & Fe42 | 13.005 | | 2 | | **0.002** | |
| Fe19 & Fe42 | 18.642 | 8 | 0.017 | Fe19 & Fe42 | 11.204 | 2 | **0.004** | Fe19 & Fe42 | 3.198 | 2 | 0.202 | | Fe19 & Fe42 |  |  | |  | | Fe19 & Fe42 | 1.736 | | 2 | | 0.420 | |
| Fe13 & Fe51 | 8.817 | 8 | 0.358 | Fe13 & Fe51 | 1.385 | 2 | 0.500 | Fe13 & Fe51 | 2.573 | 2 | 0.276 | | Fe13 & Fe51 | 0.000 | 2 | | 1.000 | | Fe13 & Fe51 | 3.754 | | 2 | | 0.153 | |
| Fe19 & Fe51 | 14.025 | 8 | 0.081 | Fe19 & Fe51 | 2.415 | 2 | 0.299 | Fe19 & Fe51 | 3.414 | 2 | 0.181 | | Fe19 & Fe51 | 0.000 | 2 | | 1.000 | | Fe19 & Fe51 | 5.597 | | 2 | | 0.061 | |
| Fe42 & Fe51 | 8.624 | 8 | 0.375 | Fe42 & Fe51 | 2.550 | 2 | 0.279 | Fe42 & Fe51 | 2.972 | 2 | 0.226 | | Fe42 & Fe51 | 0.000 | 2 | | 1.000 | | Fe42 & Fe51 | 2.302 | | 2 | | 0.316 | |
| Fe13 & Fy4 | 6.463 | 8 | 0.596 | Fe13 & Fy4 | 0.306 | 2 | 0.858 | Fe13 & Fy4 | 0.036 | 2 | 0.982 | | Fe13 & Fy4 | 1.029 | 2 | | 0.598 | | Fe13 & Fy4 | 4.839 | | 2 | | 0.089 | |
| Fe19 & Fy4 | 11.197 | 8 | 0.191 | Fe19 & Fy4 | 3.995 | 2 | 0.136 | Fe19 & Fy4 | 1.316 | 2 | 0.518 | | Fe19 & Fy4 | 1.058 | 2 | | 0.589 | | Fe19 & Fy4 | 4.282 | | 2 | | 0.118 | |
| Fe42 & Fy4 | 3.498 | 8 | 0.899 | Fe42 & Fy4 | 1.466 | 2 | 0.481 | Fe42 & Fy4 | 0.000 | 2 | 1.000 | | Fe42 & Fy4 | 0.000 | 2 | | 1.000 | | Fe42 & Fy4 | 0.111 | | 2 | | 0.946 | |
| Fe51 & Fy4 | 10.514 | 8 | 0.231 | Fe51 & Fy4 | 1.496 | 2 | 0.473 | Fe51 & Fy4 | 0.511 | 2 | 0.774 | | Fe51 & Fy4 | 0.000 | 2 | | 1.000 | | Fe51 & Fy4 | 6.678 | | 2 | | **0.035** | |
| Fe13 & Fe16 | 3.518 | 6 | 0.742 | Fe13 & Fe16 | 6.546 | 2 | **0.038** | Fe13 & Fe16 | 2.219 | 2 | 0.330 | | Fe13 & Fe16 |  |  | |  | | Fe13 & Fe16 | 0.000 | | 2 | | 1.000 | |
| Fe19 & Fe16 | 9.242 | 8 | 0.322 | Fe19 & Fe16 | 5.171 | 2 | 0.075 | Fe19 & Fe16 | 0.000 | 2 | 1.000 | | Fe19 & Fe16 |  |  | |  | | Fe19 & Fe16 | 1.800 | | 2 | | 0.407 | |
| Fe42 & Fe16 | 3.533 | 6 | 0.740 | Fe42 & Fe16 | 0.038 | 2 | 0.981 | Fe42 & Fe16 | 2.826 | 2 | 0.243 | | Fe42 & Fe16 |  |  | |  | | Fe42 & Fe16 | 0.350 | | 2 | | 0.839 | |
| Fe51 & Fe16 | 7.673 | 8 | 0.466 | Fe51 & Fe16 | 0.987 | 2 | 0.610 | Fe51 & Fe16 | 0.000 | 2 | 1.000 | | Fe51 & Fe16 | 0.000 | 2 | | 1.000 | | Fe51 & Fe16 | 5.766 | | 2 | | 0.056 | |
| Fy4 & Fe16 | 4.187 | 8 | 0.840 | Fy4 & Fe16 | 0.681 | 2 | 0.711 | Fy4 & Fe16 | 0.000 | 2 | 1.000 | | Fy4 & Fe16 | 1.398 | 2 | | 0.497 | | Fy4 & Fe16 | 2.421 | | 2 | | 0.298 | |
| Fe13 & Fy7 | 8.667 | 6 | 0.193 | Fe13 & Fy7 | 2.484 | 2 | 0.289 | Fe13 & Fy7 | 6.247 | 2 | **0.044** | | Fe13 & Fy7 |  |  | |  | | Fe13 & Fy7 | 0.019 | | 2 | | 0.991 | |
| Fe19 & Fy7 | 7.942 | 8 | 0.439 | Fe19 & Fy7 | 2.890 | 2 | 0.236 | Fe19 & Fy7 | 0.814 | 2 | 0.666 | | Fe19 & Fy7 |  |  | |  | | Fe19 & Fy7 | 3.038 | | 2 | | 0.219 | |
| Fe42 & Fy7 | 7.100 | 6 | 0.312 | Fe42 & Fy7 | 3.250 | 2 | 0.197 | Fe42 & Fy7 | 0.755 | 2 | 0.686 | | Fe42 & Fy7 |  |  | |  | | Fe42 & Fy7 | 1.198 | | 2 | | 0.549 | |
| Fe51 & Fy7 | 10.137 | 8 | 0.256 | Fe51 & Fy7 | 7.119 | 2 | **0.028** | Fe51 & Fy7 | 0.625 | 2 | 0.732 | | Fe51 & Fy7 | 0.000 | 2 | | 1.000 | | Fe51 & Fy7 | 7.321 | | 2 | | **0.026** | |
| Fy4 & Fy7 | 0.628 | 8 | 1.000 | Fy4 & Fy7 | 0.088 | 2 | 0.957 | Fy4 & Fy7 | 0.102 | 2 | 0.950 | | Fy4 & Fy7 | 0.000 | 2 | | 1.000 | | Fy4 & Fy7 | 0.442 | | 2 | | 0.802 | |
| Fe16 & Fy7 | 15.029 | 8 | 0.059 | Fe16 & Fy7 | 0.068 | 2 | 0.966 | Fe16 & Fy7 | 1.969 | 2 | 0.374 | | Fe16 & Fy7 | 0.000 | 2 | | 1.000 | | Fe16 & Fy7 | 9.826 | | 2 | | **0.007** | |
| Fe13 & Fe37 | 4.137 | 8 | 0.845 | Fe13 & Fe37 | 1.741 | 2 | 0.419 | Fe13 & Fe37 | 0.444 | 2 | 0.801 | | Fe13 & Fe37 | 0.000 | 2 | | 1.000 | | Fe13 & Fe37 | 1.870 | | 2 | | 0.393 | |
| Fe19 & Fe37 | 7.235 | 8 | 0.512 | Fe19 & Fe37 | 2.952 | 2 | 0.229 | Fe19 & Fe37 | 0.853 | 2 | 0.653 | | Fe19 & Fe37 | 2.363 | 2 | | 0.307 | | Fe19 & Fe37 | 1.198 | | 2 | | 0.549 | |
| Fe42 & Fe37 | 5.153 | 8 | 0.741 | Fe42 & Fe37 | 2.647 | 2 | 0.266 | Fe42 & Fe37 | 0.000 | 2 | 1.000 | | Fe42 & Fe37 | 2.188 | 2 | | 0.335 | | Fe42 & Fe37 | 0.619 | | 2 | | 0.734 | |
| Fe51 & Fe37 | 4.900 | 8 | 0.768 | Fe51 & Fe37 | 2.083 | 2 | 0.353 | Fe51 & Fe37 | 0.000 | 2 | 1.000 | | Fe51 & Fe37 | 0.000 | 2 | | 1.000 | | Fe51 & Fe37 | 1.125 | | 2 | | 0.570 | |
| Fy4 & Fe37 | 14.580 | 8 | 0.068 | Fy4 & Fe37 | 0.649 | 2 | 0.723 | Fy4 & Fe37 | 5.853 | 2 | 0.054 | | Fy4 & Fe37 | 1.842 | 2 | | 0.398 | | Fy4 & Fe37 | 7.511 | | 2 | | **0.023** | |
| Fe16 & Fe37 | 17.784 | 8 | **0.023** | Fe16 & Fe37 | 9.751 | 2 | **0.008** | Fe16 & Fe37 | 9.183 | 2 | **0.010** | | Fe16 & Fe37 | 0.000 | 2 | | 1.000 | | Fe16 & Fe37 | 0.000 | | 2 | | 1.000 | |
| Fy7 & Fe37 | 2.327 | 8 | 0.969 | Fy7 & Fe37 | 2.727 | 2 | 0.256 | Fy7 & Fe37 | 0.319 | 2 | 0.853 | | Fy7 & Fe37 | 0.000 | 2 | | 1.000 | | Fy7 & Fe37 | 0.065 | | 2 | | 0.968 | |
| Fe13 & P22 | 9.538 | 8 | 0.299 | Fe13 & P22 | 7.602 | 2 | **0.022** | Fe13 & P22 | 6.946 | 2 | **0.031** | | Fe13 & P22 | 0.000 | 2 | | 1.000 | | Fe13 & P22 | 0.152 | | 2 | | 0.927 | |
| Fe19 & P22 | 5.513 | 8 | 0.702 | Fe19 & P22 | 4.697 | 2 | 0.096 | Fe19 & P22 | 0.019 | 2 | 0.990 | | Fe19 & P22 | 0.000 | 2 | | 1.000 | | Fe19 & P22 | 0.443 | | 2 | | 0.801 | |
| Fe42 & P22 | 10.990 | 8 | 0.202 | Fe42 & P22 | 7.032 | 2 | **0.030** | Fe42 & P22 | 0.342 | 2 | 0.843 | | Fe42 & P22 | 0.000 | 2 | | 1.000 | | Fe42 & P22 | 3.148 | | 2 | | 0.207 | |
| Fe51 & P22 | 9.197 | 8 | 0.326 | Fe51 & P22 | 7.878 | 2 | **0.019** | Fe51 & P22 | 0.333 | 2 | 0.847 | | Fe51 & P22 | 4.556 | 2 | | 0.102 | | Fe51 & P22 | 1.338 | | 2 | | 0.512 | |
| Fy4 & P22 | 5.017 | 8 | 0.756 | Fy4 & P22 | 3.595 | 2 | 0.166 | Fy4 & P22 | 0.444 | 2 | 0.801 | | Fy4 & P22 | 0.000 | 2 | | 1.000 | | Fy4 & P22 | 1.601 | | 2 | | 0.449 | |
| Fe16 & P22 | 11.661 | 8 | 0.167 | Fe16 & P22 | 2.232 | 2 | 0.328 | Fe16 & P22 | 0.000 | 2 | 1.000 | | Fe16 & P22 | 0.000 | 2 | | 1.000 | | Fe16 & P22 | 7.877 | | 2 | | **0.019** | |
| Fy7 & P22 | ∞ | 8 | **< 0.0001** | Fy7 & P22 | ∞ | 2 | **< 0.0001** | Fy7 & P22 | 1.474 | 2 | 0.479 | | Fy7 & P22 | 0.000 | 2 | | 1.000 | | Fy7 & P22 | 7.805 | | 2 | | **0.020** | |
| Fe37 & P22 | 6.740 | 8 | 0.565 | Fe37 & P22 | 6.016 | 2 | **0.049** | Fe37 & P22 | 0.294 | 2 | 0.863 | | Fe37 & P22 | 0.000 | 2 | | 1.000 | | Fe37 & P22 | 0.648 | | 2 | | 0.723 | |
| Fe13 & Fe38 | ∞ | 8 | **< 0.0001** | Fe13 & Fe38 | 11.483 | 2 | **0.003** | Fe13 & Fe38 | 9.288 | 2 | **0.010** | | Fe13 & Fe38 | 0.000 | 2 | | 1.000 | | Fe13 & Fe38 | 0.563 | | 2 | | 0.755 | |
| Fe19 & Fe38 | 2.916 | 8 | 0.940 | Fe19 & Fe38 | 0.695 | 2 | 0.706 | Fe19 & Fe38 | 1.130 | 2 | 0.568 | | Fe19 & Fe38 | 0.000 | 2 | | 1.000 | | Fe19 & Fe38 | 1.407 | | 2 | | 0.495 | |
| Fe42 & Fe38 | 4.796 | 6 | 0.570 | Fe42 & Fe38 | 2.441 | 2 | 0.295 | Fe42 & Fe38 | 2.028 | 2 | 0.363 | | Fe42 & Fe38 |  |  | |  | | Fe42 & Fe38 | 2.095 | | 2 | | 0.351 | |
| Fe51 & Fe38 | 6.487 | 8 | 0.593 | Fe51 & Fe38 | 2.597 | 2 | 0.273 | Fe51 & Fe38 | 1.982 | 2 | 0.371 | | Fe51 & Fe38 | 0.000 | 2 | | 1.000 | | Fe51 & Fe38 | 0.192 | | 2 | | 0.908 | |
| Fy4 & Fe38 | 8.840 | 8 | 0.356 | Fy4 & Fe38 | 5.413 | 2 | 0.067 | Fy4 & Fe38 | 0.000 | 2 | 1.000 | | Fy4 & Fe38 | 1.001 | 2 | | 0.606 | | Fy4 & Fe38 | 2.879 | | 2 | | 0.237 | |
| Fe16 & Fe38 | 24.456 | 8 | **0.002** | Fe16 & Fe38 | 8.950 | 2 | **0.011** | Fe16 & Fe38 | 4.180 | 2 | 0.124 | | Fe16 & Fe38 | 4.772 | 2 | | 0.092 | | Fe16 & Fe38 | 10.725 | | 2 | | **0.005** | |
| Fy7 & Fe38 | 17.293 | 8 | **0.027** | Fy7 & Fe38 | 5.185 | 2 | 0.075 | Fy7 & Fe38 | 8.547 | 2 | **0.014** | | Fy7 & Fe38 | 0.000 | 2 | | 1.000 | | Fy7 & Fe38 | 1.420 | | 2 | | 0.492 | |
| Fe37 & Fe38 | 2.835 | 8 | 0.944 | Fe37 & Fe38 | 1.713 | 2 | 0.425 | Fe37 & Fe38 | 1.669 | 2 | 0.434 | | Fe37 & Fe38 | 0.000 | 2 | | 1.000 | | Fe37 & Fe38 | 0.000 | | 2 | | 1.000 | |
| P22 & Fe38 | 1.064 | 8 | 0.998 | P22 & Fe38 | 0.000 | 2 | 1.000 | P22 & Fe38 | 0.194 | 2 | 0.908 | | P22 & Fe38 | 0.000 | 2 | | 1.000 | | P22 & Fe38 | 0.797 | | 2 | | 0.671 | |
| Fe13 & Fl21 | 3.995 | 8 | 0.858 | Fe13 & Fl21 | 1.385 | 2 | 0.500 | Fe13 & Fl21 | 3.342 | 2 | 0.188 | | Fe13 & Fl21 | 0.000 | 2 | | 1.000 | | Fe13 & Fl21 | 0.941 | | 2 | | 0.625 | |
| Fe19 & Fl21 | 12.968 | 8 | 0.113 | Fe19 & Fl21 | 7.666 | 2 | **0.022** | Fe19 & Fl21 | 0.000 | 2 | 1.000 | | Fe19 & Fl21 | 0.000 | 2 | | 1.000 | | Fe19 & Fl21 | 2.790 | | 2 | | 0.248 | |
| Fe42 & Fl21 | 7.102 | 8 | 0.526 | Fe42 & Fl21 | 0.522 | 2 | 0.770 | Fe42 & Fl21 | 1.251 | 2 | 0.535 | | Fe42 & Fl21 | 2.177 | 2 | | 0.337 | | Fe42 & Fl21 | 5.223 | | 2 | | 0.073 | |
| Fe51 & Fl21 | 12.096 | 8 | 0.147 | Fe51 & Fl21 | 7.326 | 2 | **0.026** | Fe51 & Fl21 | 0.000 | 2 | 1.000 | | Fe51 & Fl21 | 2.910 | 2 | | 0.233 | | Fe51 & Fl21 | 2.286 | | 2 | | 0.319 | |
| Fy4 & Fl21 | 7.403 | 8 | 0.494 | Fy4 & Fl21 | 3.146 | 2 | 0.207 | Fy4 & Fl21 | 1.392 | 2 | 0.498 | | Fy4 & Fl21 | 1.010 | 2 | | 0.604 | | Fy4 & Fl21 | 1.499 | | 2 | | 0.473 | |
| Fe16 & Fl21 | 7.995 | 8 | 0.434 | Fe16 & Fl21 | 1.037 | 2 | 0.596 | Fe16 & Fl21 | 6.536 | 2 | **0.038** | | Fe16 & Fl21 | 0.000 | 2 | | 1.000 | | Fe16 & Fl21 | 0.536 | | 2 | | 0.765 | |
| Fy7 & Fl21 | 6.117 | 8 | 0.634 | Fy7 & Fl21 | 1.805 | 2 | 0.405 | Fy7 & Fl21 | 0.795 | 2 | 0.672 | | Fy7 & Fl21 | 0.000 | 2 | | 1.000 | | Fy7 & Fl21 | 3.574 | | 2 | | 0.167 | |
| Fe37 & Fl21 | 16.617 | 8 | **0.034** | Fe37 & Fl21 | 7.408 | 2 | **0.025** | Fe37 & Fl21 | 9.423 | 2 | **0.009** | | Fe37 & Fl21 | 2.406 | 2 | | 0.300 | | Fe37 & Fl21 | 1.594 | | 2 | | 0.451 | |
| P22 & Fl21 | 7.197 | 8 | 0.515 | P22 & Fl21 | 1.202 | 2 | 0.548 | P22 & Fl21 | 1.365 | 2 | 0.505 | | P22 & Fl21 | 0.000 | 2 | | 1.000 | | P22 & Fl21 | 2.442 | | 2 | | 0.295 | |
| Fe38 & Fl21 | 10.632 | 8 | 0.223 | Fe38 & Fl21 | 4.103 | 2 | 0.129 | Fe38 & Fl21 | 6.524 | 2 | **0.038** | | Fe38 & Fl21 | 0.000 | 2 | | 1.000 | | Fe38 & Fl21 | 1.118 | | 2 | | 0.572 | |
| Fe13 & Fe11 | 0.810 | 4 | 0.937 | Fe13 & Fe11 | 0.841 | 2 | 0.657 | Fe13 & Fe11 |  |  |  | | Fe13 & Fe11 |  | |  | |  | Fe13 & Fe11 | 0.000 | | 2 | | 1.000 | |
| Fe19 & Fe11 | 5.443 | 4 | 0.245 | Fe19 & Fe11 | 4.419 | 2 | 0.110 | Fe19 & Fe11 |  |  |  | | Fe19 & Fe11 |  | |  | |  | Fe19 & Fe11 | 0.526 | | 2 | | 0.769 | |
| Fe42 & Fe11 | 0.714 | 4 | 0.950 | Fe42 & Fe11 | 0.058 | 2 | 0.971 | Fe42 & Fe11 |  |  |  | | Fe42 & Fe11 |  | |  | |  | Fe42 & Fe11 | 0.624 | | 2 | | 0.732 | |
| Fe51 & Fe11 | 6.816 | 4 | 0.146 | Fe51 & Fe11 | 1.136 | 2 | 0.567 | Fe51 & Fe11 |  |  |  | | Fe51 & Fe11 |  | |  | |  | Fe51 & Fe11 | 5.178 | | 2 | | 0.075 | |
| Fy4 & Fe11 | 2.362 | 4 | 0.670 | Fy4 & Fe11 | 1.788 | 2 | 0.409 | Fy4 & Fe11 |  |  |  | | Fy4 & Fe11 |  | |  | |  | Fy4 & Fe11 | 0.000 | | 2 | | 1.000 | |
| Fe16 & Fe11 | 2.945 | 2 | 0.229 | Fe16 & Fe11 | 2.651 | 2 | 0.266 | Fe16 & Fe11 |  |  |  | | Fe16 & Fe11 |  | |  | |  | Fe16 & Fe11 |  | |  | |  | |
| Fy7 & Fe11 | 14.785 | 4 | **0.005** | Fy7 & Fe11 | 3.941 | 2 | 0.139 | Fy7 & Fe11 |  |  |  | | Fy7 & Fe11 |  | |  | |  | Fy7 & Fe11 | 9.286 | | 2 | | **0.010** | |
| Fe37 & Fe11 | 3.881 | 4 | 0.422 | Fe37 & Fe11 | 4.697 | 2 | 0.096 | Fe37 & Fe11 |  |  |  | | Fe37 & Fe11 |  | |  | |  | Fe37 & Fe11 | 0.000 | | 2 | | 1.000 | |
| P22 & Fe11 | 7.291 | 4 | 0.121 | P22 & Fe11 | 1.974 | 2 | 0.373 | P22 & Fe11 |  |  |  | | P22 & Fe11 |  | |  | |  | P22 & Fe11 | 5.597 | | 2 | | 0.061 | |
| Fe38 & Fe11 | 1.621 | 4 | 0.805 | Fe38 & Fe11 | 1.360 | 2 | 0.507 | Fe38 & Fe11 |  |  |  | | Fe38 & Fe11 |  | |  | |  | Fe38 & Fe11 | 0.000 | | 2 | | 1.000 | |
| Fl21 & Fe11 | 7.215 | 4 | 0.125 | Fl21 & Fe11 | 8.556 | 2 | **0.014** | Fl21 & Fe11 |  |  |  | | Fl21 & Fe11 |  | |  | |  | Fl21 & Fe11 | 0.000 | | 2 | | 1.000 | |
| Fe13 & Fe21 | 1.608 | 2 | 0.448 | Fe13 & Fe21 | 2.637 | 2 | 0.268 | Fe13 & Fe21 |  |  |  | | Fe13 & Fe21 |  | |  | |  | Fe13 & Fe21 |  | | |  |  | |
| Fe19 & Fe21 | 5.665 | 2 | 0.059 | Fe19 & Fe21 | 6.712 | 2 | **0.035** | Fe19 & Fe21 |  |  |  | | Fe19 & Fe21 |  | |  | |  | Fe19 & Fe21 |  | | |  |  | |
| Fe42 & Fe21 | 3.244 | 2 | 0.198 | Fe42 & Fe21 | 3.243 | 2 | 0.198 | Fe42 & Fe21 |  |  |  | | Fe42 & Fe21 |  | |  | |  | Fe42 & Fe21 |  | | |  |  | |
| Fe51 & Fe21 | 0.367 | 2 | 0.832 | Fe51 & Fe21 | 0.354 | 2 | 0.838 | Fe51 & Fe21 |  |  |  | | Fe51 & Fe21 |  | |  | |  | Fe51 & Fe21 |  | | |  |  | |
| Fy4 & Fe21 | 1.948 | 2 | 0.377 | Fy4 & Fe21 | 1.958 | 2 | 0.376 | Fy4 & Fe21 |  |  |  | | Fy4 & Fe21 |  | |  | |  | Fy4 & Fe21 |  | | |  |  | |
| Fe16 & Fe21 | 3.667 | 2 | 0.160 | Fe16 & Fe21 | 3.176 | 2 | 0.204 | Fe16 & Fe21 |  |  |  | | Fe16 & Fe21 |  | |  | |  | Fe16 & Fe21 |  | | |  |  | |
| Fy7 & Fe21 | 0.021 | 2 | 0.990 | Fy7 & Fe21 | 0.096 | 2 | 0.953 | Fy7 & Fe21 |  |  |  | | Fy7 & Fe21 |  | |  | |  | Fy7 & Fe21 |  | | |  |  | |
| Fe37 & Fe21 | 2.237 | 2 | 0.327 | Fe37 & Fe21 | 2.769 | 2 | 0.251 | Fe37 & Fe21 |  |  |  | | Fe37 & Fe21 |  | |  | |  | Fe37 & Fe21 |  | | |  | |  |
| P22 & Fe21 | 1.747 | 2 | 0.417 | P22 & Fe21 | 1.902 | 2 | 0.386 | P22 & Fe21 |  |  |  | | P22 & Fe21 |  | |  | |  | P22 & Fe21 |  | | |  | |  |
| Fe38 & Fe21 | 0.003 | 2 | 0.998 | Fe38 & Fe21 | 0.002 | 2 | 0.999 | Fe38 & Fe21 |  |  |  | | Fe38 & Fe21 |  | |  | |  | Fe38 & Fe21 |  | | |  | |  |
| Fl21 & Fe21 | 0.477 | 2 | 0.788 | Fl21 & Fe21 | 0.407 | 2 | 0.816 | Fl21 & Fe21 |  |  |  | | Fl21 & Fe21 |  | |  | |  | Fl21 & Fe21 |  | | |  | |  |
| Fe11 & Fe21 | 2.097 | 2 | 0.350 | Fe11 & Fe21 | 2.085 | 2 | 0.353 | Fe11 & Fe21 |  |  |  | | Fe11 & Fe21 |  | |  | |  | Fe11 & Fe21 |  | | |  | |  |

**Supplementary table 6**

Output table from STRUCTURE HARVESTER.

| K | Mean LnP(K) | Stdev LnP(K) | Ln'(K) | \|Ln''(K)\| | DeltaK |
| --- | --- | --- | --- | --- | --- |
| 1 | -9545,9 | 0,3 | NA | NA | NA |
| 2 | -9378,8 | 1,5 | 167,0 | 152,9 | 99,9 |
| 3 | -9364,7 | 16,7 | 14,1 | 67,3 | 4,0 |
| 4 | -9283,3 | 20,8 | 81,4 | 43,4 | 2,1 |
| 5 | -9245,3 | 38,3 | 38,0 | 277,0 | 7,2 |
| 6 | -9484,4 | 137,5 | -239,0 | 482,1 | 3,5 |
| 7 | -9241,3 | 66,7 | 243,1 | 485,2 | 7,3 |
| 8 | -9483,3 | 29,2 | -242,1 | 123,8 | 4,2 |
| 9 | -9601,6 | 92,0 | -118,3 | 55,7 | 0,6 |
| 10 | -9775,6 | 165,5 | -174,0 | 60,7 | 0,4 |
| 11 | -9888,8 | 170,4 | -113,3 | 53,7 | 0,3 |
| 12 | -10055,9 | 182,4 | -167,0 | 74,6 | 0,4 |
| 13 | -10148,3 | 145,8 | -92,4 | 112,3 | 0,8 |
| 14 | -10353,1 | 173,3 | -204,8 | 113,2 | 0,7 |
| 15 | -10444,6 | 151,5 | -91,6 | NA | NA |
| Delta K values estimated with the Evanno method (2005) | | | | | |
